# Effects of PKM2 on global metabolic changes and prognosis in hepatocellular carcinoma: from gene expression to drug discovery

**DOI:** 10.1186/s12885-018-5023-0

**Published:** 2018-11-21

**Authors:** Wen-Wen Lv, Dahai Liu, Xing-Cun Liu, Tie-Nan Feng, Lei Li, Bi-Yun Qian, Wen-Xing Li

**Affiliations:** 1 Hongqiao International Institute of Medicine, Shanghai Tongren Hospital/Faculty of Public Health, Shanghai Jiao Tong University School of Medicine, Shanghai, 200025 China; 2 Clinical Research Center, Shanghai Jiao Tong University School of Medicine, Shanghai, 200025 China; 3 School of Stomatology and Medicine, Foshan University, Foshan, 528000 Guangdong China; 4 Department of Gastrointestinal Surgery, The First Affiliated Hospital of Anhui Medical University, Hefei, 230032 Anhui China; 5 Key Laboratory of Animal Models and Human Disease Mechanisms, Kunming Institute of Zoology, Chinese Academy of Sciences, Kunming, 650223 Yunnan China; 6 Kunming College of Life Science, University of Chinese Academy of Sciences, Kunming, 650204 Yunnan China

**Keywords:** Hepatocellular carcinoma, PKM2, Metabolic, Overall survival, Drugs

## Abstract

**Background:**

Hepatocellular carcinoma (HCC) is a malignant tumor that threatens global human health. High PKM2 expression is widely reported in multiple cancers, especially in HCC. This study aimed to explore the effects of PKM2 on global gene expression, metabolic damages, patient prognosis, and multiple transcriptional regulation relationships, as well as to identify several key metabolic genes and screen some small-molecule drugs.

**Methods:**

Transcriptome and clinical HCC data were downloaded from the NIH-GDC repository. Information regarding the metabolic genes and subsystems was collected from the Recon 2 human metabolic model. Drug-protein interaction data were obtained from the DrugBank and UniProt databases. We defined patients with PKM2 expression levels ≥11.25 as the high-PKM2 group, and those with low PKM2 expression (< 11.25) were defined as the low-PKM2 group.

**Results:**

The results showed that the global metabolic gene expression levels were obviously divided into the high- or low-PKM2 groups. In addition, a greater number of affected metabolic subsystems were observed in the high-PKM2 group. Furthermore, we identified 98 PKM2-correlated deregulated metabolic genes that were associated with poor overall patient survival. Together, these findings suggest more comprehensive influences of PKM2 on HCC. In addition, we screened several small-molecule drugs that target these metabolic enzymes, some of which have been used in antitumor clinical studies.

**Conclusions:**

HCC patients with high PKM2 expression showed more severe metabolic damage, transcriptional regulation imbalance and poor prognosis than low-PKM2 individuals. We believe that our study provides valuable information for pathology research and drug development for HCC.

**Electronic supplementary material:**

The online version of this article (10.1186/s12885-018-5023-0) contains supplementary material, which is available to authorized users.

## Background

Hepatocellular carcinoma (HCC) is one of the two major forms of primary liver cancer and accounts for 85–90% of all primary liver cancers [1]. HCC is typically caused by viral hepatitis infection or fatty liver disease. Cryptogenic cirrhosis, which is frequently linked with nonalcoholic steatohepatitis (NASH) / nonalcoholic fatty liver disease (NAFLD) in patients with metabolic syndrome, diabetes and obesity, is an increasingly significant cause of HCC [2]. Furthermore, metabolic syndrome itself is also a strong risk factor for HCC [3]. Increasing evidence suggests that diabetes is an independent risk factor for HCC [4], particularly diabetes controlled by diet, insulin or sulfonylureas [5]. Additionally, a series of metabolic alterations in HCC, including elevated glycolysis, gluconeogenesis, and b-oxidation levels and reduced tricarboxylic acid cycle and D-12 desaturase levels, were observed [6].

Pyruvate kinase (PK) catalyzes the last and physiologically irreversible step in glycolysis, the conversion of phosphoenol pyruvate (PEP) to pyruvate via the transfer of a phosphate group to ADP [7]. In mammals, four PK isoforms exist that are encoded by two genes. The PKLR gene encodes PKL and PKR [8]. The former is exclusive to erythrocytes, and the latter is expressed primarily in the liver, with low expression in the kidney. The PKM1 and PKM2 isoforms are encoded by PKM through alternative splicing of the mutually exclusive exons 9 and 10, which generate 56-amino acid regions that differ at 22 residues [7, 8]. PKM1 is mainly expressed in brain, heart, and muscle tissue, whereas PKM2 is expressed in most tumor cells, embryonic tissue and many adult tissues, including the kidney, spleen, lung and intestine [7, 9]. PKM2 plays a central role in maintaining the metabolism program of cancer cells and other proliferating cell types and is over-expressed in a broad range of human cancers [7].

High PKM2 expression is correlated with poor prognosis for patients with multiple types of solid tumors compared with the prognosis of patients with low PKM2 expression levels [10, 11]. Our previous study also found high PKM2 expression is independently associated with poor overall survival in HCC patients [12]. The knockdown of PKM2 in HCC cells inhibited cell proliferation and induced apoptosis in vitro and in vivo [13]. Furthermore, PKM2 plays critical in Warburg effect, gene expression, cell cycle progression and many other fundamental cellular functions and is highlighted as an important integrator of diverse cellular stimuli to modulate metabolic flux and cancer cell proliferation [14]. Recent studies found that PKM2 is the dominant form highly expressed in HCC and is a direct target of miR-122, which serves as a prognostic biomarker and induces apoptosis and growth arrest by downregulating PKM2 in HCC [15, 16].

Establishment of a human metabolic model provided considerable data support and systematic analytical methods for human metabolic disease research [17]. In this study, we obtained information regarding all human metabolic genes and metabolic subsystems from the Recon2 human metabolic model [18]. Considering that HCC is a metabolic disease, we aimed to explore the associations between PKM2 expression and overall metabolic changes in HCC, identify several risk metabolic genes and discover some drugs.

## Methods

### Data collection

HCC RNA-seq and clinical data were downloaded from the NIH-GDC repository (https://portal.gdc.cancer.gov/) and cBioPortal (http://www.cbioportal.org/) databases [19]. In total, we downloaded TCGA level 3 data that included 371 primary hepatocellular carcinoma patients and 50 controls. All samples were analyzed by Illumina HiSeq 2000 RNA Sequencing Version 2. RNA-seq by expectation-maximization (RSEM) expression values were used for statistical analysis. We divided all HCC samples into two groups according to their log2-converted median expression of PKM2 (11.25). Case samples with PKM2 expression ≥11.25 comprised the high-PKM2 group, and those with low PKM2 expression (< 11.25) comprised the low-PKM2 group. PKM2 copy number data were downloaded from the cBioPortal database and PKM2 promoter methylation data were obtained from MethHC (http://methhc.mbc.nctu.edu.tw/php/search.php?opt=gene). The methylation data contains 204 HCC patients and 49 controls, and the copy number data contains 364 HCC patients but no controls. We mapped these samples to the transcriptome data and compared the difference among controls, low-PKM2 patients and high-PKM2 patients. The validation datasets of GSE14520 (contributed by Roessler S, including 225 patients) and GSE25097 (contributed by Tung EK, including 268 patients) were downloaded from NCBI-GEO (https://www.ncbi.nlm.nih.gov/geo/). We used these two datasets to verify the effect of PKM2 on global metabolic gene expression.

### Preprocessing and differential expression analysis

R statistical software v3.3.3 was used to perform data preprocessing. We removed genes with median expression values = 0 in all patients. In total, we filtered 16706 genes in 371 HCC patients and 50 controls. All expression values were log2-transformed. Human metabolic genes were extracted from the Recon 2 human metabolism model (https://www.vmh.life/). This model contains 1789 unique genes that belong to 99 metabolic subsystems. We mapped 1492 metabolic genes and 95 subsystems in our HCC data. Differentially expressed gene analysis between HCC patients and controls in the high- and low-PKM2 groups was performed using the empirical Bayes algorithm (function “eBayes”) in the “limma” package [20]. Differences (up- or down-regulated) were considered statistically significant for absolute value log2-transformed fold-changes ≥1 and false discovery rate (FDR) adjusted *P* values ≤0.05.

### Metabolic subsystem enrichment and correlation analysis

We used javaGSEA desktop application v3.0 (http://software.broadinstitute.org/gsea/index.jsp) [21] to perform gene set enrichment analysis (GSEA) of the mapped metabolic subsystems in high-PKM2 vs. controls, low-PKM2 vs. controls and high-PKM2 vs. low PKM2. Gene sets with less than 10 genes or more than 500 genes were excluded. The t-statistic mean of the genes was computed in each metabolic subsystem using a permutation test with 1000 replications. Subsystems with normalized enrichment scores (NESs) > 0 were considered up-regulated, and subsystems with NESs < 0 were considered down-regulated. Statistical significance was identified as *P* values ≤0.05. A univariate correlation model was used to analyze correlation between PKM2 and other metabolic genes in high- and low-PKM2 patients. Genes with absolute correlation coefficient values ≥0.5 and *P* values ≤0.05 were considered significant.

### Single gene and gene interaction survival analyses

We used Venn diagrams to show the overlapping genes among the up-regulated and PKM2-positively correlated genes as well as the down-regulated and PKM2-negatively correlated genes in the high- and low-PKM2 groups. All survival analyses were conducted using “survival” package in R. A Cox proportional hazards model was used to estimate the independent effects of these overlapping genes on the total overall survival of the HCC patients. Each gene was divided into two groups according to its median expression. Patients with gene expression levels less than the median comprised the low-expression group, and those with gene expression levels higher than the median comprised the high-expression group. The reference group of up-regulated genes was defined as patients in the low-expression group, and the reference group of down-regulated genes was defined as patients in the high-expression group. According to our grouping, genes with hazard ratios (HRs) > 1 were considered risk factors. Conversely, genes with HRs < 1 were considered protective factors. To explore the effect of interactions between PKM2 and metabolic genes on patients’ overall survival, we used Cox proportional hazards model to perform interaction survival analysis of PKM2 and metabolic genes that uncorrelated with PKM2. We screened these uncorrelated genes that were not affect patients’ prognosis alone and no expression difference in high- or low-PKM2 patients compared to controls. The “simPH” package [22] in R was used to simulate and plot quantities of interactions from Cox proportional hazard models. The Kaplan-Meier curves were used to show the difference of these interactions on patients’ survival. A *P* value ≤0.05 was considered as significant.

### Transcriptional regulatory network analysis

Using the analysis described above, we defined overlapping genes with survival differences as risk metabolic genes. We used the TRRUST v2.0 web server [23] to find the transcription factors (TFs) targeting these genes. TRRUST v2.0 is a manually curated database of human and mouse transcriptional regulatory networks. It contains 9,996 TF-target regulatory relationships for 824 human TFs and provides information regarding the regulation type (such as activation or repression) between the queried TFs and target genes (http://www.grnpedia.org/trrust/). All these relationships were derived from PubMed articles. We used these TFs and risk metabolic genes to construct transcriptional regulatory networks (TRNs) in high- and low-PKM2 groups. Because the transcriptional regulation information of multiple risk metabolic genes is not recorded in the database, the co-expression method was used to construct TRNs. The correlation coefficients of these TFs and risk metabolic genes were calculated in each group, and the TF-target pairs with absolute value of correlation coefficients ≥0.5 and FDR *P*-values ≤0.05 were selected to construct the TRN.

### Construction of the risk metabolic genes and drug network

We used these risk metabolic genes and drugs that target these proteins to construct the network. Information regarding the drugs and targeting proteins was downloaded from the DrugBank database (https://www.drugbank.ca/) [24]. Because the records in the DrugBank database are protein IDs and drugs interactions, we used UniProt (http://www.uniprot.org/) to find the protein IDs corresponding to these risk metabolic genes. By combining the DrugBank and UniProt databases, we obtained 14834 gene-protein-drug interactions in total, including 2127 unique genes and 6255 unique drugs. We extracted the interactions of risk metabolic genes and drugs and used Cytoscape v3.4.0 to visualize the network.

## Results

### Global metabolic gene expression levels were different in the high- and low-PKM2 groups

**Fig. 1:**
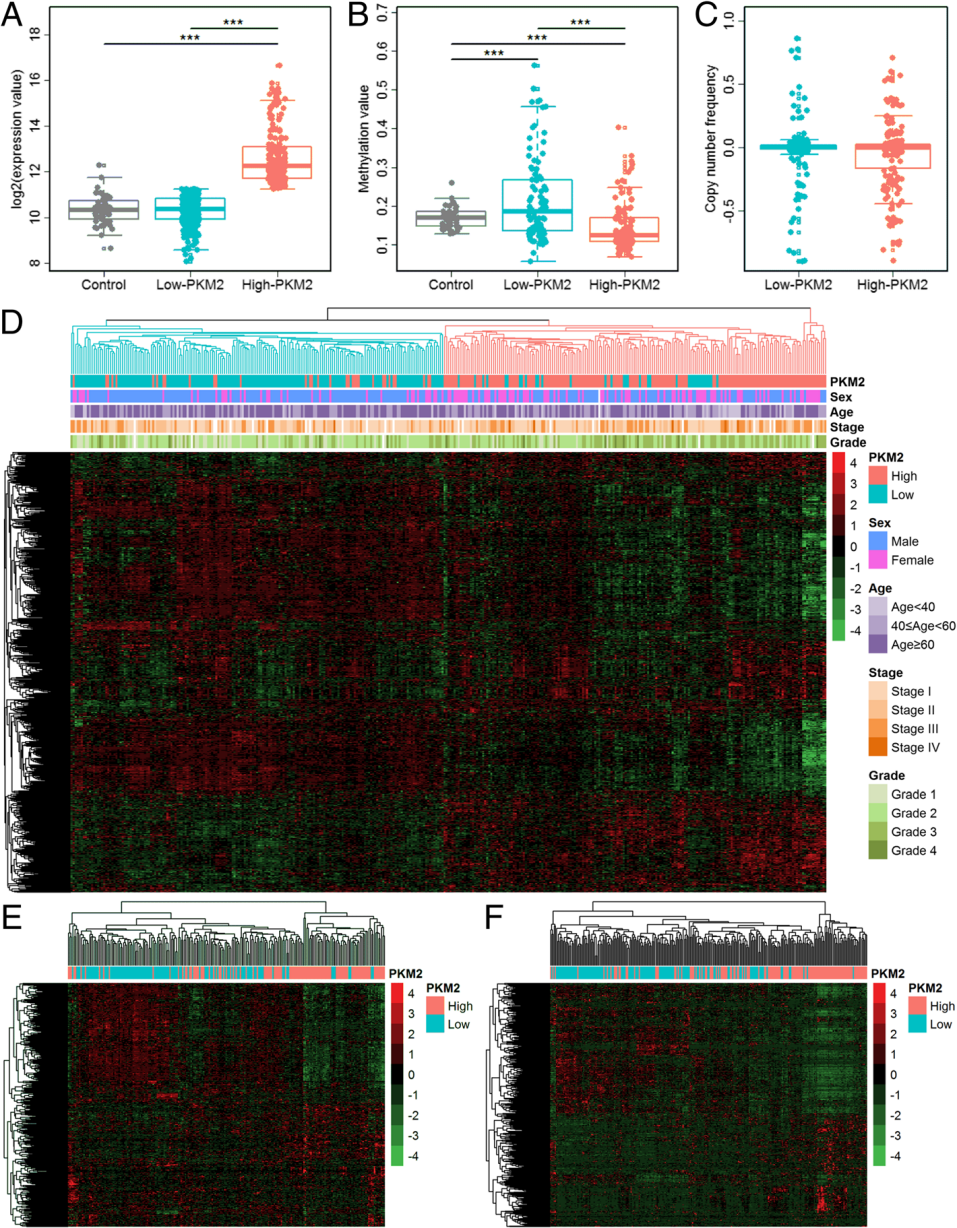
PKM2 and metabolic gene expression profiles. **a** The expression levels of PKM2 in low-PKM2 (< 11.25) patients (light blue) were similar to the controls (gray), whereas the expression levels of PKM2 in high-PKM2 (≥ 11.25) patients (red) were significantly higher than the controls. **b** Methylation value of PKM2 in low-PKM2 patients (light blue) were higher than the controls (gray), whereas the methylation value of PKM2 in high-PKM2 patients (red) were significantly lower than the controls and the low-PKM2 patients. **c** There was no difference of copy number variation between high- and low-PKM2 patients. **d** Heatmap of 1492 metabolic genes in 371 patients (TCGA data). The hierarchical clustering results suggested the expression levels of global metabolic genes were divided into clustering group I (blue) and clustering group II (red). Statistical difference of PKM2 expression, sex, age, grade and stage between the two groups showed in Additional file 1: Tables S3 and S4. **e** Heatmap of 1319 metabolic genes in 225 patients (GSE14520, validation dataset 1). **f** Heatmap of 1490 metabolic genes in 268 patients (GSE25097, validation dataset 2). These two hierarchical clustering results validated the expression profiles of metabolic genes were different between high- and low-PKM2 patients. Statistical significance: * *P* < 0.05, ** *P* < 0.01, *** *P* < 0.001

We calculated differentially expressed genes in high- and low-PKM2 patients (Additional file 1: Tables S1 and S2). In total, 4521 and 3314 deregulated genes were identified in high- and low-PKM2 patients, respectively. Figure 1 showed the global metabolic gene expression levels in the high- and low-PKM2 groups. The expression of PKM2 in low-PKM2 patients was similar to that of the controls; however, the expression of PKM2 in high-PKM2 patients was significantly higher than that of the controls (Fig. 1a). Furthermore, the methylation value of PKM2 in low-PKM2 patients were higher than the controls, whereas the methylation value of PKM2 in high-PKM2 patients were significantly lower than the controls and the low-PKM2 patients (Fig. 1b). However, there was no difference of copy number variation between high- and low-PKM2 patients (Fig. 1c). These results suggested that the PKM2 expression was consistent with the methylation status. The expression profiles of 1492 metabolic genes in 371 HCC patients showed in Fig. 1d. Through hierarchical clustering, the expression levels of global metabolic genes were divided into two groups. We compared the PKM2 expression, sex, age, stage and grade between the two clustering groups and found most of patients with low-PKM2 expression were in clustering group I and patients with high-PKM2 expression were mainly in clustering group II. This significant difference is independent with other clinical variables (Additional file 1: Tables S3 and S4). Furthermore, the hierarchical clustering results of other two validation datasets (GSE14520 and GSE25097) showed similar trends (Fig. 1e and f). All these results suggested that PKM2 was correlated with the expression of global metabolic genes.

### Patients in the high-PKM2 group showed more severe metabolic abnormalities

**Fig. 2:**
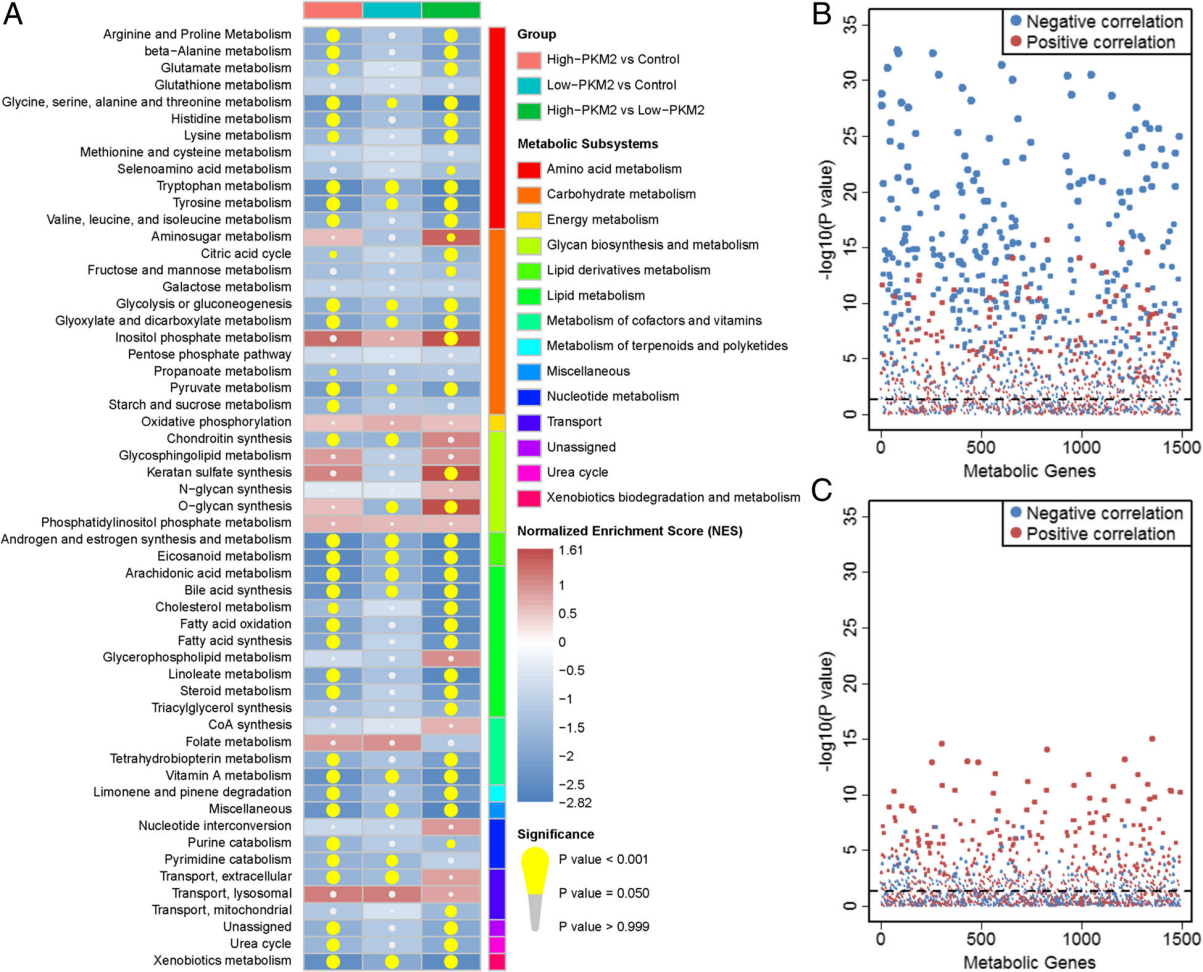
Metabolic subsystem enrichment results and correlation between PKM2 and metabolic genes. **a** Metabolic subsystem enrichment analysis of high-PKM2 vs. controls, low-PKM2 vs. controls and high-PKM2 vs. low-PKM2. The red box represents the metabolic subsystem is up-regulated and the blue box represents the metabolic subsystem is down-regulated. The yellow circle indicates the metabolic subsystem is significantly enriched. **b** Correlation of PKM2 and metabolic genes in the high-PKM2 group. **c** Correlation of PKM2 and metabolic genes in the low-PKM2 group. The red points represent positive correlations, and the blue points represent negative correlations. The absolute values of the correlation coefficients are represented by the size of the points. The horizontal dashed line shows the significant level (*P* ≤ 0.05)

**Fig. 3:**
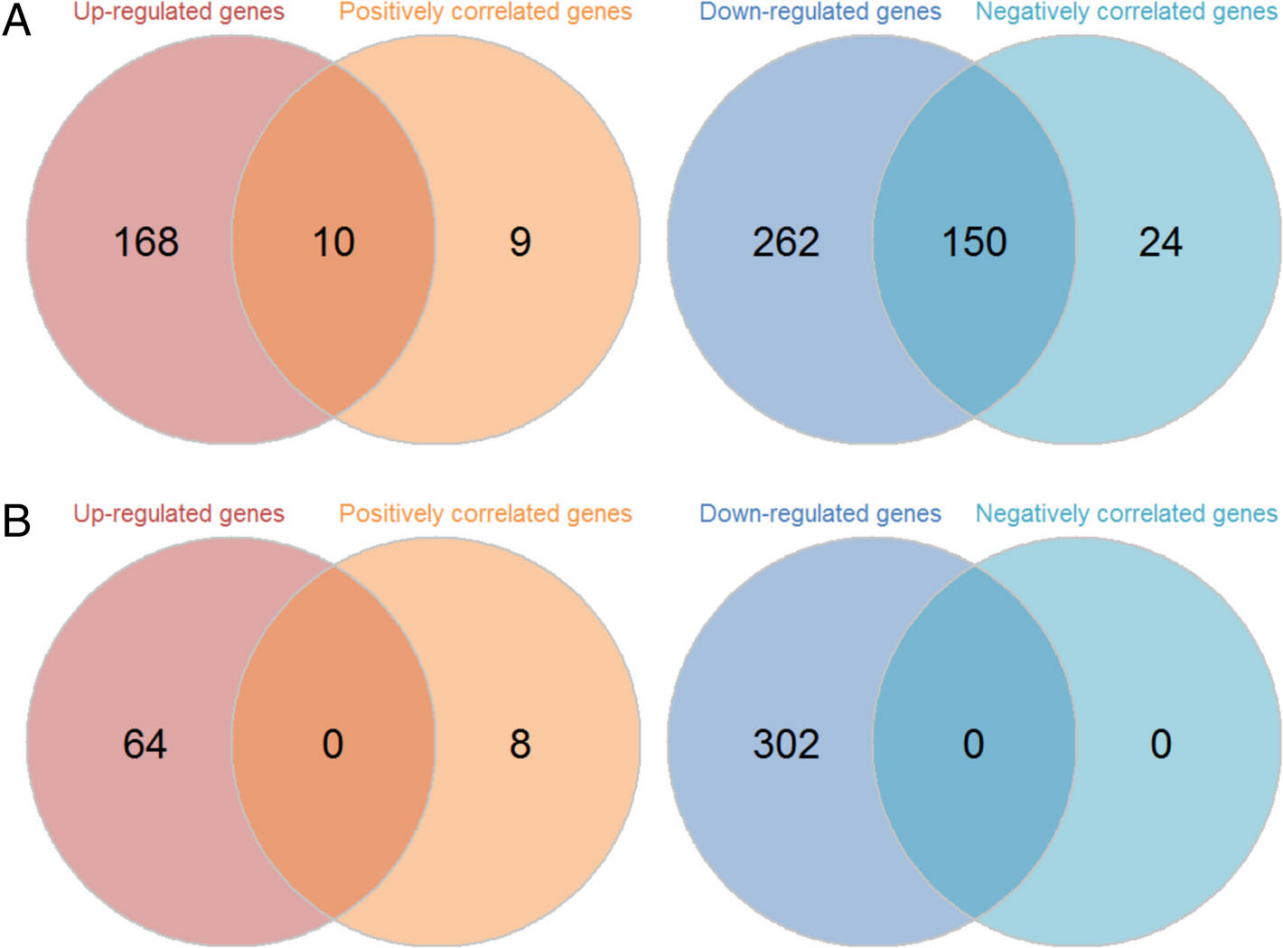
Venn diagram of deregulated and PKM2-correlated genes in the high-PKM2 (**a**) and low-PKM2 (**b**) groups. The left panel shows the Venn diagram of up-regulated and PKM2-positively correlated genes, and the right panel shows the Venn diagram of down-regulated and PKM2-negatively correlated genes

The enriched metabolic subsystems in high-PKM2 vs. controls, low-PKM2 vs. controls and high-PKM2 vs. low-PKM2 showed in Fig. 2a. The original results for GSEA showed in Additional file 1: Table S5. There were 35 down-regulated metabolic subsystems in high-PKM2 vs. controls, whereas only 17 down-regulated metabolic subsystems in low-PKM2 vs. controls. No up-regulated metabolic subsystem was found in high-PKM2 vs. controls or low-PKM2 vs. controls. Furthermore, there were 4 up-regulated and 34 down-regulated metabolic subsystems in high-PKM2 vs. low-PKM2. There have similar trends of down-regulated metabolic subsystems in high-PKM2 vs. controls and high-PKM2 vs. low-PKM2. The results showed that most of these subsystems were associated with amino acid metabolism, carbohydrate metabolism, lipid and lipid derivatives metabolism. Correlations between PKM2 and other metabolic genes in the high- and low-PKM2 groups showed in Fig. 2b and c, respectively. Details of the correlation coefficients and *P* values in all the metabolic genes in the two groups listed in Additional file 1: Tables S6 and S7. According to our screening criteria, 19 PKM2-positively correlated genes and 174 PKM2-negatively correlated genes were identified in the high-PKM2 group. However, only 8 PKM2-positively correlated genes and no PKM2-negatively correlated genes were observed in the low-PKM2 group. We further screened common genes between up-regulated and PKM2-positively correlated genes and between down-regulated and PKM2-negatively correlated genes in the high- and low-PKM2 groups (Fig. 3). The results showed that 160 common genes existed in the high-PKM2 group. However, no common genes were observed in the low-PKM2 group. These results suggested that patients with high PKM2 expression suffer more serious metabolic damage compared with that of low-PKM2 patients.

### PKM2-correlated deregulated genes associated with poor overall survival

**Fig. 4:**
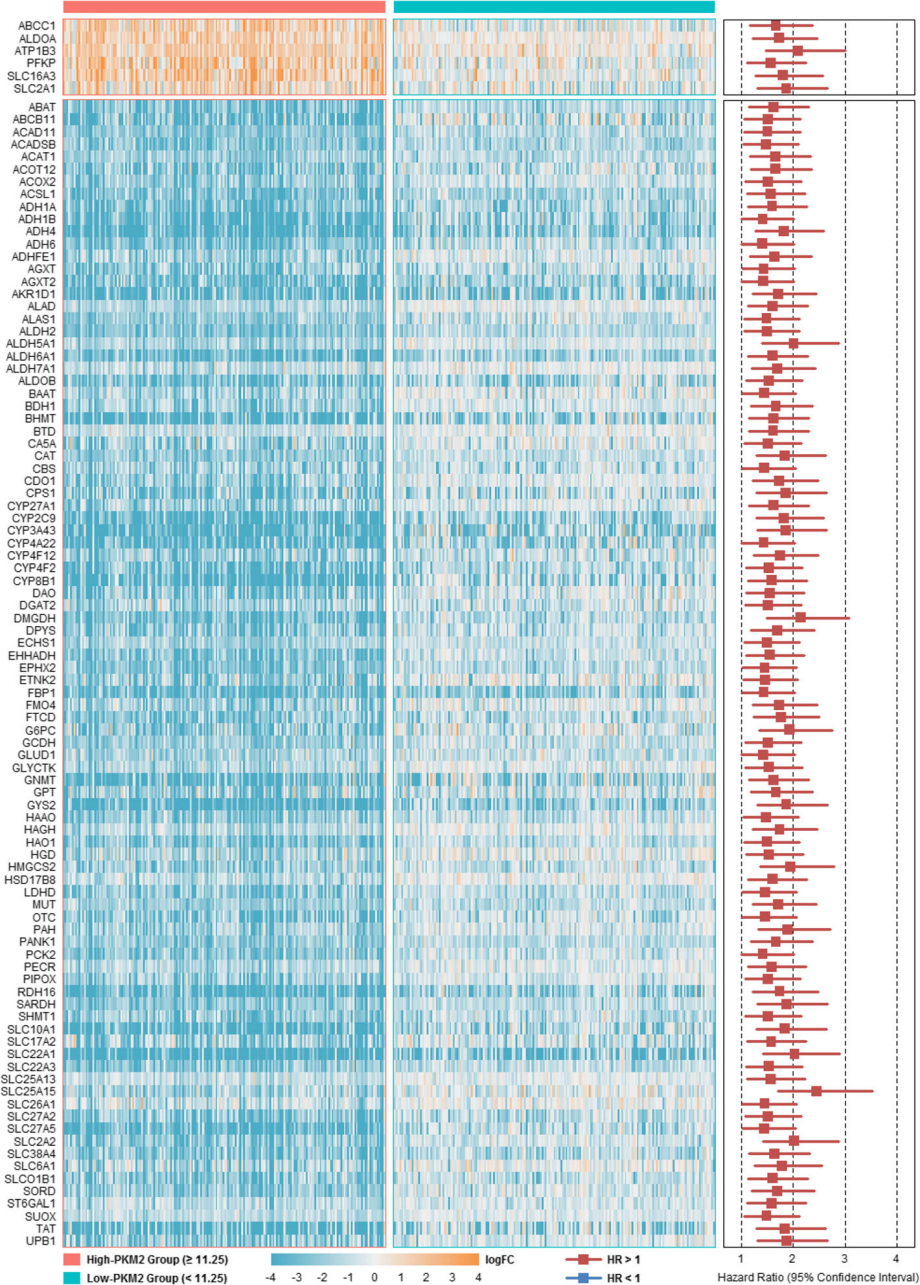
Personalized expression and survival analysis of PKM2-correlated deregulated genes. The heatmap shows the personalized log2 fold changes of these genes in the high-PKM2 (≥ 11.25) and low-PKM2 (< 11.25) groups. The personalized fold changes were calculated as the gene expression values for each patient/mean expression of the gene in controls. For survival analysis, the reference group of up-regulated genes was defined as patients with levels below the median expression level of the gene, and the reference group of down-regulated genes was defined as patients with levels higher than the median expression level of the gene

**Fig. 5:**
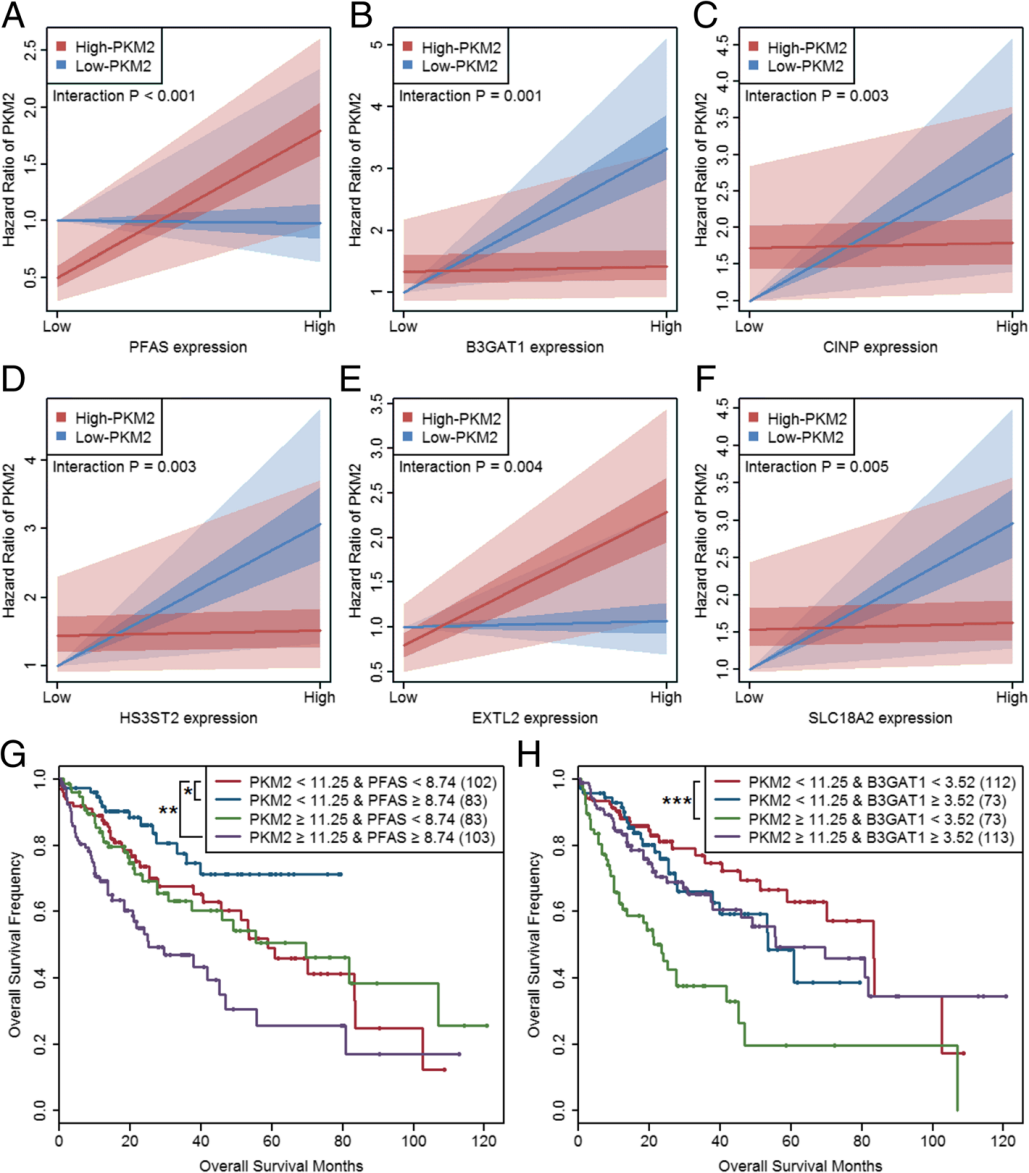
Interaction survival analysis of PKM2 and PKM2-uncorrelated metabolic genes. **a** Quantities of interaction between PKM2 and PFAS on patients’ overall survival. **b** Quantities of interaction between PKM2 and B3GAT1 on patients’ overall survival. **c** Quantities of interaction between PKM2 and CINP on patients’ overall survival. **d** Quantities of interaction between PKM2 and HS3ST2 on patients’ overall survival. **e** Quantities of interaction between PKM2 and EXTL2 on patients’ overall survival. **f** Quantities of interaction between PKM2 and SLC18A2 on patients’ overall survival. **g** Kaplan-Meier curves of PKM2 and PFAS on patients’ overall survival. **h** Kaplan-Meier curves of PKM2 and B3GAT1 on patients’ overall survival. All these PKM2-uncorrelated genes showed no expression difference in high- or low-PKM2 groups. The figure showed top six interactions sorted by interaction *P* values. Details see in Additional file 1: Table S10. Statistical significance: * *P* < 0.05, ** *P* < 0.01, *** *P* < 0.001

We performed survival analysis using the above mentioned 160 PKM2-correlated deregulated genes in all HCC patients (Additional file 1: Tables S8 and S9). Among these 160 genes, 98 genes (including 6 up-regulated genes and 92 down-regulated genes) were significantly correlated with overall patient survival. Therefore, we classified these 98 genes as risk metabolic genes, which were deregulated and had survival risks in all patients. Figure 4 shows the personalized fold changes (The gene expression values for each patient/mean expression of the gene in controls) in the high- and low-PKM2 groups as well as the survival risks of these 98 genes. Interestingly, these genes all showed worse overall survival in high-PKM2 patients than in low-PKM2 patients. Furthermore, we screened 462 metabolic genes that was no correlation with PKM2, and these genes showed no expression or survival difference in HCC patients. We performed interactive survival analysis of these uncorrelated metabolic genes with PKM2 (Additional file 1: Table S10). The results showed that there were 36 significantly interactions (interaction *P* < 0.05). We screened top 6 interactions based on the decreasing order of interaction *P* values and plotted the quantities of interactions (Fig. 5a-f). The Kaplan-Meier curves of PKM2 and uncorrelated metabolic genes also showed significance (Fig. 5g-h). These results suggested that the interactions of PKM2 with metabolic genes have significant effects on patients’ overall survival.

### Transcriptional regulatory networks of risk metabolic genes were related to PKM2 expression

**Fig. 6:**
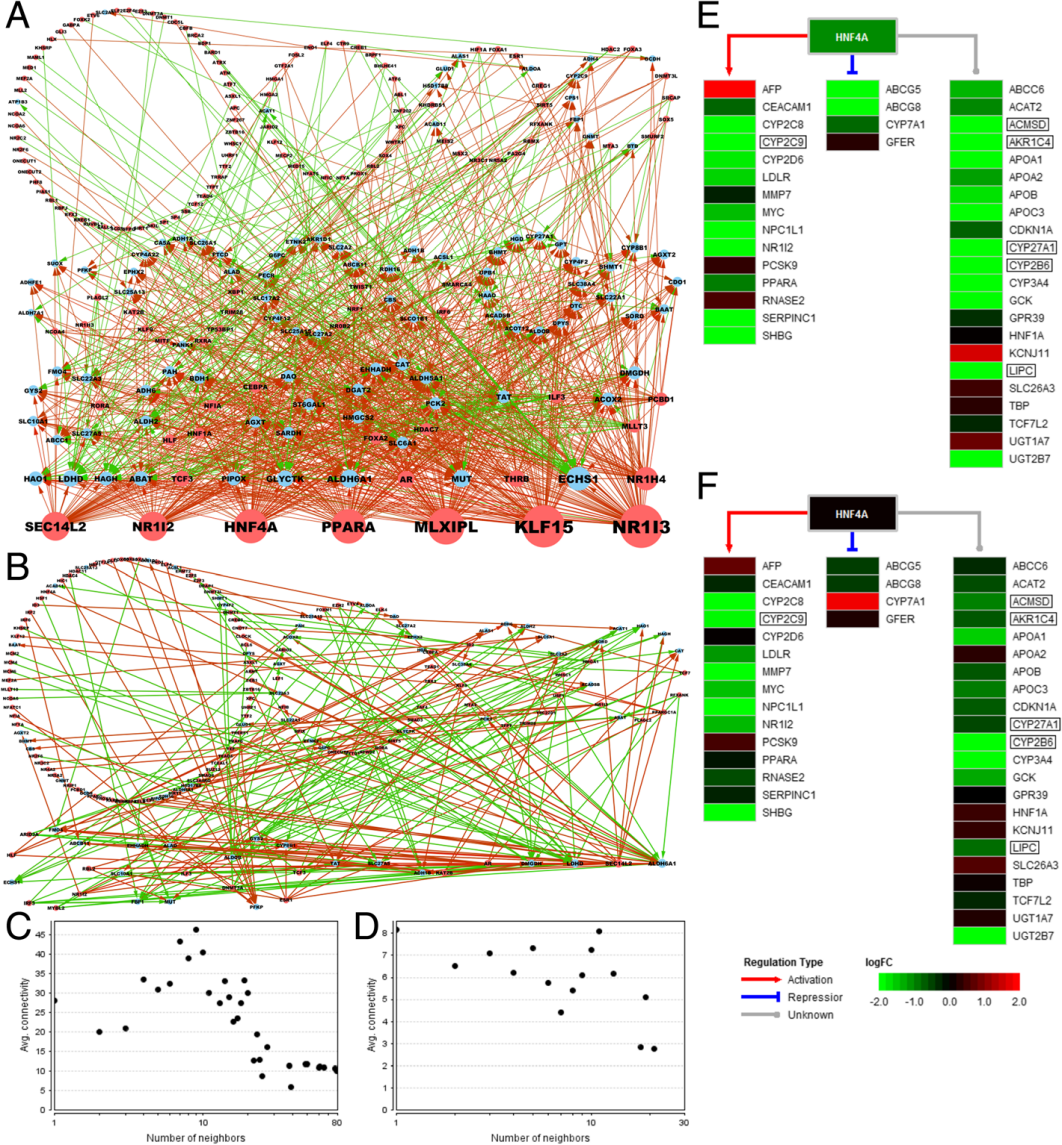
Transcriptional regulation relationships in high- and low-PKM2 groups. **a** Transcriptional regulatory network of risk metabolic genes in high-PKM2 group. **b** Transcriptional regulatory network of risk metabolic genes in low-PKM2 group. The red circle represents transcription factors and the blue circle represents target genes. The red line represents positive correlation and the green line represents negative correlation. The size of the circle indicates the number of nodes. **c** Neighborhood connectivity in high-PKM2 group. **d** Neighborhood connectivity in low-PKM2 group. **e** HNF4A and its target genes in high-PKM2 group. **f** HNF4A and its target genes in low-PKM2 group. The gradient color from red to green is expressed as the logFC value of each gene. The red, blue and gray lines show the type of HNF4A regulation on its targets. PKM2-correlated and survival risk genes are shown in the box

Transcriptional regulatory relationships of TFs and the risk metabolic genes in high- and low-PKM2 group were showed in Fig. 6a and b. There were 232 nodes in the high-PKM2 group and 175 nodes in the low-PKM2 group. The average number of neighbors is 8.267 in the high-PKM2 group and 2.857 in the low-PKM2 group. Furthermore, the neighborhood connectivity in high-PKM2 group were higher than low-PKM2 group (Fig. 6c and d). From the perspective of network connectivity and complexity, the transcriptional regulation relationships of TFs and risk metabolic genes were stronger in the high-PKM2 group than in the low-PKM2 group. There were only 47 documented regulation relationships contain 23 TFs and 29 risk metabolic genes (Additional file 1: Table S11). In these TF-target interactions, HNF4A regulated the most target genes. We displayed the expression profiles and regulation relationships of HNF4A and its target genes (including risk metabolic genes and other genes) in the high-PKM2 and low-PKM2 groups (Fig. 6e and f). In total, 6 risk metabolic genes were regulated by HNF4A (CYP2C9 is activated by HNF4A, and the regulation types of the other 5 genes are unknown). In high-PKM2 patients, HNF4A was expressed at low levels, and its target genes were all significantly down-regulated. However, in low-PKM2 patients, HNF4A expression was not altered, and four HNF4A-regulated risk metabolic genes also showed no significant difference, although these genes exhibited low expression.

### Network of risk metabolic genes and drugs that target these genes

**Fig. 7:**
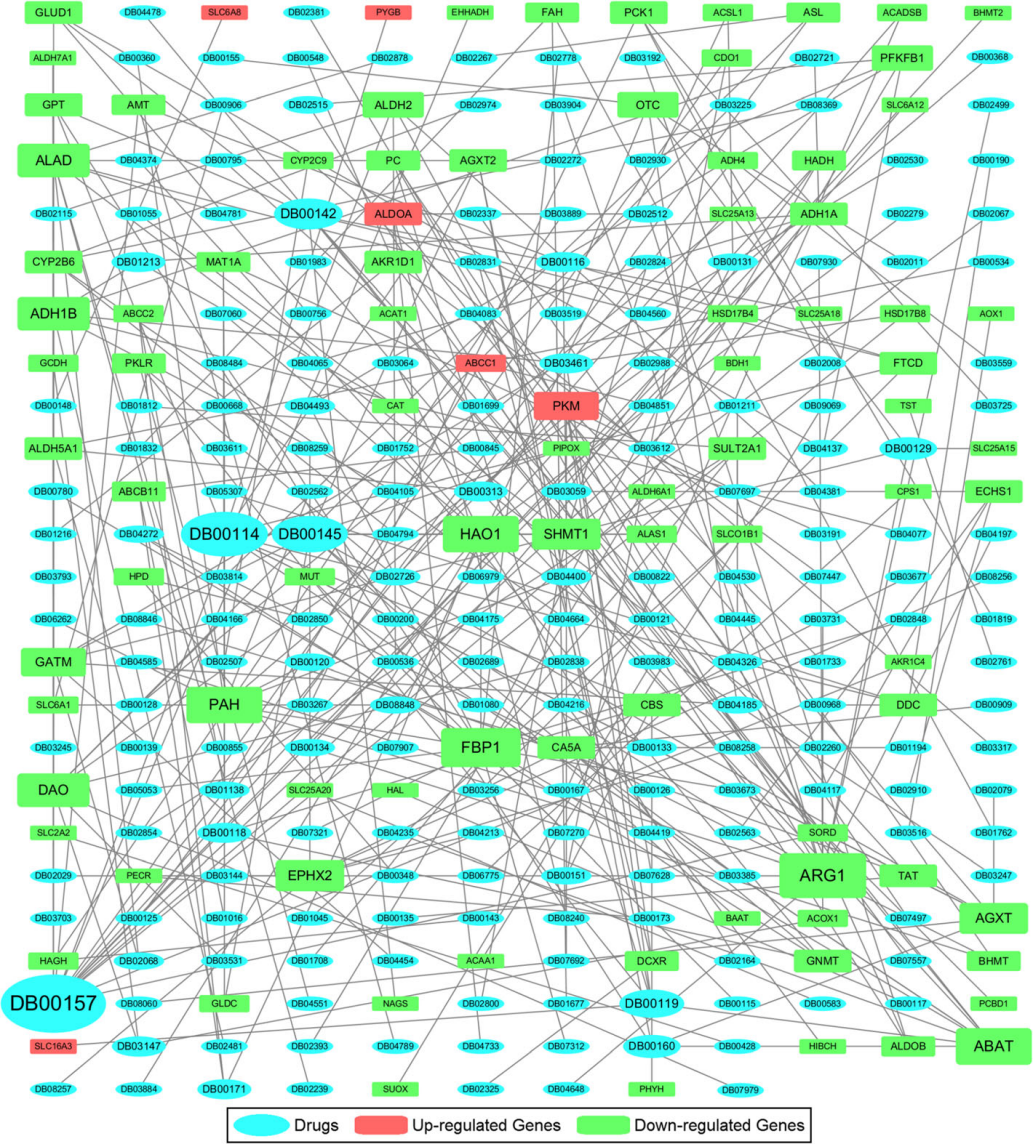
Network of metabolic target genes and drugs. Genes in the network were correlated with PKM2 and survival risk. The network connected genes and drugs if their records were in the DrugBank database. The red rectangle, green rectangle and light blue ellipse represent up-regulated genes, down-regulated genes and drugs, respectively. The node size is proportional to the number of connections

We obtained 290 interactions of the risk metabolic genes, proteins and drugs (Additional file 1: Table S12). The network of drugs and risk metabolic genes showed in Fig. 7. The network included 7 up-regulated metabolic genes (PKM was searched in the DrugBank database, and no records of its subtypes exist), 86 down-regulated metabolic genes and 204 drugs. In total, 12 enzymes interacted with more than 5 drugs. Among these proteins, ARG1 binds 12 drugs, and FBP1 binds 10 drugs. ABAT, HAO1 and PAH each bind 9 drugs; ADH1B, ALAD and DAO each bind 8 drugs; AGXT, EPHX2 and SHMT1 each bind 7 drugs; and GATM and PKM each bind 6 drugs. In addition, 5 drugs interacted with more than 5 enzymes. These drugs were pyridoxal phosphate, pyruvic acid, L-glutamic acid, glycine and NADH. We noticed that most of these drugs are coenzymes or substrates of the risk metabolic enzymes. Considering the numerous down-regulated metabolic subsystems in HCC patients, we hypothesized that supplements of these drugs may enhance the activity of metabolic reactions and improve the disease. We also used L1000CDS2 (Characteristic Direction Signature Search) web tool (http://amp.pharm.mssm.edu/L1000CDS2/#/index) to search the compounds that perturb these risk metabolic genes. The input parameter is the list of 6 up-regulated risk metabolic genes and 92 down-regulated risk metabolic genes. The predicted results showed there were 35 unique compounds that may perturb these genes in different cell lines (Additional file 1: Table S13). However, only two compounds were recorded in DrugBank database and did not appear in our results. Due to large differences between human tissues and cell lines, and many compounds used in the Library of Integrated Cellular Signatures (LINCS) program were not record in DrugBank database, we believe that there should be more detailed research to prove these results in the future.

## Discussion

This study found that patients expressing PKM2 at high levels exhibited abnormal expression of whole metabolic genes, detrimental metabolic subsystems changes, unfavorable prognoses, and transcriptional regulation imbalances compared with low-PKM2 patients. In addition, we identified 98 risk metabolic genes that were significantly correlated with PKM2. Utilizing the DrugBank and UniProt databases, we screened 204 drugs that target these genes.

HCC is metabolically different from normal liver tissue in many ways, including glycolysis, lipid metabolism, the TCA cycle, amino acid metabolism, and several PPAR signaling pathways [25]. In addition, close relationships have been observed among metabolic syndrome, nonalcoholic fatty liver disease, and liver cancer [26]. In the American population, metabolic syndrome occurrences were significantly higher in HCC (37.1%) and intrahepatic cholangiocarcinoma (ICC) (29.7%) patients than in the normal group (17.1%, *P* < 1E-4) [3]. Furthermore, metabolic syndrome is not only a risk factor for HCC in elderly people, but it also promotes cancer in people younger than 65 years [27]. A prospective study of 578,700 adults suggested that high BMI, blood glucose and composite metabolic syndrome z-scores were positively associated with liver cancer risks [28]. Another study in a Swedish population also drew a similar conclusion that metabolic syndrome components were strongly associated with primary liver cancer [29]. In a normal liver, glucose is phosphorylated to glucose-6-phosphate (G6P) by glucokinase (GCK). Normally, G6P undergoes glycolysis and is converted to pyruvate before going through the citric acid cycle in mitochondria to generate ATP through oxidative phosphorylation [25]. However, liver tumor cells produce ATP by aerobic glycolysis, called the Warburg effect, which is accompanied by increased glycolysis and decreased oxidative phosphorylation. In our study, we observed many genes that were over-expressed in glycolysis (including HKDC1, HK2, PFKP, ENO2, ALDOA, GPD2, G6PC3, BPGM, etc.) in high-PKM2 patients. By contrast, more genes were down-regulated in gluconeogenesis, resulting in negative normalized enrichment scores (NESs) in the GSEA results. Pyruvate kinase is activated by its substrate and inhibited by high levels of ATP. In tumors, low enzymatic activity of PKM2 favors the Warburg effect. In addition, high PKM2 mRNA expression was associated with tumor growth and poor outcomes [25]. In our study, patients with high PKM2 expression showed more severe metabolic abnormalities and poorer overall survival than low-PKM2 patients. Furthermore, many down-regulated metabolic genes were significantly negatively correlated with PKM2 in the high-PKM2 group. These results suggest a pleiotropic effect of PKM2 on HCC.

Our study observed that 98 PKM2-correlated genes had a certain relationship with prognosis. The expression statuses of these genes in high-PKM2 patients were linked to poor survival, and some have been widely reported. Solute carrier family 2 member 1 (SLC2A1), also known as GLUT1, encodes a major glucose transporter in the blood-brain barrier. Studies showed that GLUT1 is over-expressed in HCC and suggested GLUT1 as a potential target [25]. In our study, high GLUT1 expression was independently associated with poor overall survival, and its interaction with PKM2 exacerbated this consequence. SLC16A3, belonging to the proton-linked monocarboxylate transporter (MCT) family, was significantly up-regulated in liver cancer in multiple datasets [30] and in our study. Deregulation of this gene may influence intracellular acidic environments [31]. Our study showed that high levels of SLC16A3 and PKM2 expression interactively led to poor prognosis. Dimethylglycine dehydrogenase (DMGDH) was expressed at low levels, negatively correlated with PKM2 and correlated with low patient survival in this study. Decreased DMGDH levels in liver tumor cells and the effects of DMGDH over-expression on the suppression of migration, invasion and metastasis has already been proven. Thus, DMGDH is suggested to be a potential diagnostic and prognostic marker for HCC [32]. SLC22A1, encoding organic cation transporter-1 (OCT1), was expressed at low levels, associated with worse HCC patient survival and significantly associated with advanced HCC stages [33]. Furthermore, a previous study showed that HCC and cholangiocarcinoma development is accompanied by the appearance of aberrant SLC22A1 variants. Thus, aberrant SLC22A1 variants together with its low expression may dramatically affect the ability of sorafenib to reach active intracellular concentrations in HCC tumors [34]. In this study, we also observed that decreased SLC22A1 expression was associated with poor prognosis, and its interaction effect with PKM2 further reduced patient survival. In addition, this study identified ATP1B3, SLC25A15 and other key metabolic genes that were deregulated in HCC and associated with patient overall survival. However, these genes have not yet been reported. Given that these genes were correlated with PKM2, we believe that a more complex mechanism of PKM2 and metabolic genes may exist in HCC and needs to be further studied.

The transcriptional regulation relationships of these key metabolic genes showed a relatively large difference between the high- and low-PKM2 groups, especially HNF4A and its target genes. Hepatocyte nuclear factor 4 alpha (HNF4A) is a nuclear transcription factor that binds DNA as a homodimer, and it has distinct transcriptional regulatory mechanisms in human liver and brain tissues [35]. Similar to our findings, HNF4A and several HNF4A target genes were down-regulated in liver cancer patients [35]. Suppression of HNF4A in mouse livers showed profound influences on zonated metabolic functions, cell proliferation and oncogenesis [36]. An HNF4A-microRNA-194/192 signaling axis was suggested to influence glucose metabolism, cell adhesion and migration, tumorigenesis and tumor progression, as well as epigenetic regulation in a recent study [37]. In our study, HNF4A transcriptionally regulated LIPC, ACMSD, AKR1C4, CYP27A1 and other metabolic genes and showed differential expression in the high- and low-PKM2 groups. In addition, FOS, MYCN, POU5F1 and other TFs also showed differential expression and regulation in high- and low-PKM2 patients. Combined with previous reports, we speculate that the carcinogenic effects of these TFs may be influenced by PKM2 expression in HCC.

High PKM2 expression in liver tumor cells is accompanied by a decrease in total cellular pyruvate kinase activity. Notably, elevated PKM2 enzyme activity may compromise both its proanabolic and antioxidant functions and shows an anti-cancer effect in HCC [38]. Therefore, the use of small-molecule PKM2 activators may be an appropriate approach to disrupt cancer cell metabolism for therapeutic purposes [38], and PKM2’s substrate may be a good target choice [39]. Many of the drugs screened in this study are essential human metabolites. Although some of these supplements may promote cancer cell growth, many studies also reported that metabolites supplement have a tumor suppressor effect. A previous study reported that S-adenosylmethionine (SAMe) can selectively induce Bcl-xS that promotes apoptosis in liver cancer cells [40]. Aminooxyacetic acid (AOAA) is a cystathionine-β-synthase (CBS) inhibitor, studies showed it suppresses the proliferation of colon cancer cells in vitro and reduces tumor growth in vivo [41, 42]. Another study suggested that hexachlorophene cause the growth arrest of B lymphoma cells by reducing the expression of cyclin-D1 and c-Myc [43]. Furthermore, studies reported that nontoxic compound disulfiram (DSF) targeted tumor cellular copper inhibited of the proteasomal activity, resulting in tumor cells apoptosis induction [44]. The compounds screened in this study contain the above anti-cancer activity drugs, therefore, we believe that these screened compounds may help to liver cancer treatment.

## Conclusions

In summary, this study showed several unfavorable effects of high PKM2 expression on whole gene expression, metabolic functions, prognosis and transcriptional regulation relationships in HCC patients. Therefore, we recommend PKM2 and PKM2-correlated risk genes can be used as biomarkers or therapeutic targets of HCC. In addition, we screened several drugs that target with these risk genes. This study pointed complex regulatory mechanisms of PKM2 and other risk metabolic genes on HCC, and the potential carcinogenic mechanisms require further study.

## Additional file

Additional file 1: **Table S1.** Differentially expressed genes in high-PKM2 group. **Table S2.** Differentially expressed genes in low-PKM2 group. **Table S3.** Descriptive statistics of PKM2 and other clinical features grouped by hierarchical clustering. **Table S4.** Multivariate logistic regression between PKM2 and hierarchical clustering groups with adjusted covariates. **Table S5.** Details of gene set enrichment analysis in high-PKM2 vs. controls, low-PKM2 vs. controls and high-PKM2 vs. low-PKM2. **Table S6.** Correlation between PKM2 and metabolic genes in high-PKM2 group. **Table S7.** Correlation between PKM2 and metabolic genes in low-PKM2 group. **Table S8.** Survival analysis of up-regulated and PKM2-positively correlated metabolic genes. **Table S9.** Survival analysis of down-regulated and PKM2-negatively correlated metabolic genes. **Table S10.** Interaction of PKM2 and PKM2-uncorrelated metabolic genes on patients’ overall survival. **Table S11.** Transcriptional regulation relationships of key metabolic genes. **Table S12.** Information of key metabolic genes and drugs. **Table S13.** Predicted compounds that affect risk metabolic genes by L1000CDS2 web tools. (XLSX 794 kb)

## Abbreviations

FDR: False discovery rate
GSEA: Gene set enrichment analysis
HCC: Hepatocellular carcinoma
HR: Hazard ratio
NES: Normalized enrichment score
PK: Pyruvate kinase
RSEM: RNA-seq by expectation-maximization
TCGA: The Cancer Genome Atlas
TF: Transcription factor
TRN: Transcriptional regulatory network
